# 

**Published:** 1911-09-16

**Authors:** 


### GIFTS.

The Surgical Aid Society has received a donation of £10 from Mrs. M. E. V. Reilly.

Mr. John Hawe, of Norfolk Road, Brighton, has left £200 to the Cancer Hospital, Fulham Road.

The Rev. George Stott, of West Barnet, Middlesex, has left £10 to the Lying-in Charity of St. Michael's, Coventry.

Miss Frances S. Brodrick, of Eaton Terrace, has left £700 for the endowment of a bed in the St. Barnabas Ward of St. Andrew's Hospital, Clewer.

Mr. George Mathwin, of Newcastle-on-Tyne, coal exporter, has left £1,000 to the Royal Victoria Infirmary, Newcastle.

Mrs. Maria Goolden, of Orme Court, Bayswater, has bequeathed £500 to the Cancer Hospital, Brompton, and £500 to the Imperial Cancer Research Fund.

Mrs. Margaret Nisbet Millar, of Helensburgh, has left £1,250 to the Royal Hospital for Sick Children, Edinburgh, for the purpose of endowing a cot in memory of her daughter.

Mr. Henry Hayman has bequeathed £100 each to the London Hospital, the Jews' Hospital and Orphan Asylum, Norwood, and the Hospital for Jewish Incurables, South Tottenham; also £500 to the Jewish Board of Guardians.

Sir Henry Howe Bemrose, of Lonsdale Hill, Derby, formerly M.P. for Derby, has bequeathed £50 each to the Derbyshire Royal Infirmary, the Children's Hospital, Derby, and to the Matlock Convalescent Home.

Mrs. Eliza Seilliere Moss, of Aigburth, Liverpool, has left £100 each to the Royal Southern Hospital, Liverpool, the Children's Infirmary, the David Lewis Northern Hospital, the Home for Incurables, the Bluecoat Hospital, and the Queen Victoria District Nursing Society.

Mr. Lesser Lesser, of Westbourne Terrace, W., has left £200 to the London Hospital, £100 each to St. Mary's Hospital and the Middlesex Hospital. He also left £200 to the Jewish Board of Guardians, and £100 each to the Hospital and Home for Jewish Incurables, and to the Jews' Hospital and Orphan Asylum.

Under the will of the late Mr. John Thomas Johnson, ironmaster, of West Bromwich, the following institutions will receive £1,000 each: The Birmingham General Hospital, the Queen's Hospital, Birmingham, the West Bromwich Hospital, the Walsall Cottage Hospital, and the Wolverhampton Orphan Asylum.

Mr. Richard Smith, of Birmingham, licensed victualler, who died last July, has left, subject to the life interest of his wife, a sum of £1,250 to the Birmingham General Hospital to endow a bed which is to be preferentially used for the benefit of persons who are or have been professional acrobats, athletes, or gymnasts. He also left £250 each to the Queen's Hospital, Birmingham, the Women's Hospital, Sparkbrook, the Birmingham and Midland Eye Hospital, and the Birmingham and Midland Free Hospital for Sick Children.

"The Prescriber."—A year's subscription to the "Prescriber," which is a most interesting and instructive monthly devoted to topics of treatment, is an investment which every general practitioner should hazard. Those who decide to send in their subscriptions—10s. 6d. for the year—during the present month will receive a copy of the new B.P. Codex 1911, an entirely revised and rearranged work, which should be on the shelves of every doctor's library.

### APPOINTMENTS VACANT.

**Full particulars of these vacancies can be obtained on reference to our advertisement columns:**

Cornwall County Asylum, Bodmin.—Third Assistant Medical Officer.

Coventry and Warwickshire Hospital.—Junior House Surgeon.

Guest Hospital, Dudley.—Assistant House Surgeon.

Leicester Infirmary.—**House Physician.**

North Riding Infirmary, Middlesbrough.—Assistant House Surgeon.

Queen's Hospital for Children.—House Surgeon.

Royal Surrey County Hospital, Guildford.—Assistant House Surgeon.

Seamen's Hospital Society, Greenwich.—Two House Physicians and two House Surgeons.

Walsall and District Hospital.—House Physician and Casualty Officer.

Wolverhampton General Hospital.—House Surgeon.

